# *Drosophila* USP5 Controls the Activation of Apoptosis and the Jun N-Terminal Kinase Pathway during Eye Development

**DOI:** 10.1371/journal.pone.0092250

**Published:** 2014-03-18

**Authors:** Xiaolan Fan, Qinzhu Huang, Xiaolei Ye, Yi Lin, Yuting Chen, Xinhua Lin, Jia Qu

**Affiliations:** School of Optometry and Ophthalmology and Eye Hospital, Wenzhou Medical University, Wenzhou, Zhejiang, China; University College London, United Kingdom

## Abstract

The Jun N-terminal kinase pathway plays an important role in inducing programmed cell death (apoptosis) and is activated in a variety of contexts. The deubiquitinating enzymes (DUBs) are proteases regulating the protein stability by ubiquitin-proteasome system. Here, for the first time, we report the phenotypes observed during eye development that are induced by deleting *Drosophila* USP5 gene, which encodes one of the USP subfamily of DUBs. *usp5* mutants displayed defects in photoreceptor differentiation. Using genetic epistasis analysis and molecular markers, we show that most of these phenotypes are caused by the activation of apoptosis and JNK pathway. These data may provide a mechanistic model for understanding the mammalian *usp5* gene.

## Introduction

The establishment and maintenance of animal homeostasis depends on the balance between cell death and cell proliferation. Programmed cell death, or apoptosis, is a key physiological process that is conserved between nematodes, insects, and vertebrates, which is used to eliminate cells[1]. Caspases are a family of cysteine proteases (cysteinyl aspartate-specific proteases) that play a crucial role in apoptosis [1], [2], [3]: they destroy vital cellular proteins and thus cause cell death.

Caspases are present in all animal cells; however, their activity is suppressed by the inhibitor of apoptosis proteins (IAPs) [4], [5], [6]. In *Drosophila*, the proapoptotic genes *reaper* (*rpr*), *grim*, and *head involution defective* (*hid*) have been identified as activators of apoptotic machinery within cells[7]. Their products bind to the *Drosophila* IAP-1 (DIAP-1), which leads to the release of caspases. One target of DIAP1 is the caspase-9 ortholog DRONC (*Drosophila* NEDD2-like caspase) [8]. There are two tandem repeats known as the Baculovirus IAP Repeats (BIR) and the RING domain at the carboxyl terminus of DIAP1 [9]. The BIR domains are necessary for binding with caspases [10], [11], [12]; however, binding of DIAP1 with DRONC is not sufficient for inhibition of DRONC as ubiquitination of DRONC is required to regulate its apoptotic activity [13]. The RING domain of DIAP1 provides the E3-ubiquitin ligase activity that is required for ubiquitination of the target proteins [6], [14]. DIAP1-mediated ubiquitination of DRONC does not lead to its degradation by proteasomes; rather, ubiquitination directs the activation of DRONC [13].

The Jun N-terminal kinase (JNK) pathway has been identified as one of the major proapoptotic factors in *Drosophila* and vertebrates. JNK activates *hid* and *rpr* genes [15], [16], which in turn initiate the cell death process. JNK not only functions upstream of the activation of propoptotic genes, but also downstream of DRONC to initiate secondary activation of additional proapoptotic genes[17].

Ubiquitination is an enzymatic process in which proteins are modified by the 76-amino acid protein ubiquitin. Ubiquitination is important not only for protein degradation, but also for the regulation of protein function, such as protein trafficking and protein interactions [18], [19], [20]. The ubiquitination process is reversible. DUBs are proteases that process ubiquitin or ubiquitin-like gene products, remodel polyubiquitin-chains on target proteins, and reverse the modification of proteins by a single ubiquitin protein[21]. It has recently been shown that knock-down of the deubiquitinating enzyme USP5 (isopeptidaseT) causes p53 activation [22]. USP5 is involved in the disassembly of free polyubiquitin by removing ubiquitin from the proximal end of unanchored polyubiquitin chains [23], [24]. When USP5 is knocked-down, unanchored polyubiquitin accumulates. Inhibition of proteasomal degradation of p53, without a defect in p53 ubiquitination, is consistent with a mechanism involving the competition of free polyubiquitin with ubiquitinated p53 for proteasomal recognition.

It is unknown if USP5 is involved in ocular development or in the regulation of other signaling pathways. To explore USP5 function, we generated an *usp5* null allele and characterized the *usp5* mutant phenotype during eye development. The *usp5* mutant contained a reduced number of photoreceptors (R8, R3/R4) and cone cells compared to wild type. Genetic interaction analysis indicated that *usp5* regulates apoptosis and the JNK pathway during eye development, consistent with its proposed role as a negative regulator of apoptosis and the JNK pathway.

## Materials and Methods

### *Drosophila* genetics

All stocks were maintained and crossed at 25 °C according to standard procedures. The en-Gal4, ap-Gal4/BCG, ey-Gal4, yw,hs-flp;hs-CD8-GFPFRT2A/Tm6B, ywflp;M3LhsGFPFRT2A/Tm6B,yw;Δ2-3/Tm6B,yw,hs-flp;Act>y+>Gal4-UAS-CD8-GFP/Cyo,yw,hs-flp;act-Gal4-UASGFP/Cyo;Gal80FRT2A/Tm6B provided by Xinhua Lin lab. yw; P{EPgy2}CG12082EY23569,yw;P{EPgy2}CG12082EY20760/TM3,mirr-Gal4;puc-lacZ/Tm6B,UAS-p35 got out from bloomington. Usp5-RNAi (VDRC#17567, 17568) we obtained from VDRC.

### *Usp5* knockout flies

The *usp5* knockout flies were generated using “P-element Hopping” technology. The details described as this paper[25]. The sequence of the primers used to PCR the *usp5* coding sequence was: 5′-GACAGGTCGCTTAATAGTGTGACC-3′;

3′-CATCCCAGACTTCCAGCAGC-5′. The deletion DNA sequence was 1.673 kb include the UBP and part of the UCH domain.

### Generation of clones

Mutant clones in the eye discs were generated by the *FLP–FRT* method [26], [27]. The fly was heat-shocked at 37°C for 2 h in first and second instars larval to induce clones. The disc was dissected at third instar stage. The *usp5* mutant clones were marked by the absence of GFP.

Mosaics Analysis with a Repressible Cell Marker (MARCM) clones were induced in first and second instar larvae by heat-shock for 2 h at 37°C. The disc was dissected at third instar stage. Expression of *UAS*-*p35* in *usp5* mosaic discs was accomplished by *act*- *Gal4*.

### Polyclonal Antibodies

A fragment of the *usp5* cDNA encoding the amino acids 226–515 was amplified by PCR using primers that inserted a 5′in-frame EcoRI site and a 3′NotI site. This fragment was cloned into the EcoR I and NoT I sites of the pGEX-4T-1 vector and used to produce the GST fusion protein to immunize mice.

### Imunohistochemistry

Eye imaginal discs from the indicated larval and pupal stage were dissected at third instar stage. Fixation and antibody staining of eye imaginal discs were performed as described [28]. Primary antibodies were used as the following dilutions: Rat anti-Elav 1∶50 (Developmental Studies Hybridoma Bank, DSHB); Mouse anti-ubiquitin, Lys48 specific 1∶500 (Millipore); Rabbit anti-Spalt 1∶200 (made in our laboratory); Mouse anti-Cut 1∶50 (DSHB); Guinea pig anti-Sens 1∶200 (made in our laboratory); Mouse anti-USP5 1∶800; Rabbit anti HID 1∶10 (Santa Cruz); Mouse anti-DIAP1 1∶5was generously provided by Dr. Bruce A. Hay (California Institute of Technology); Rabbit anti-cleaved Caspase-3 1∶100 (Cell Signaling Technology, Danvers, MA, USA); Mouse anti-pJNK1∶150 (Cell Signaling Technology, Danvers, MA, USA); Chicken anti-β-gal 1∶1000 (Abcam). The primary antibodies were detected by fluorescent-conjugated secondary antibodies (Invitrogen). We used the Zeiss 710 laser confocal microscopy to observe the samples and take images.

### Scanning electron microscopy

Adult flies were anesthetized, mounted on stages and observed under a scanning election microscope, the VE-7800 (keyence, Tokyo, Japan) in the low-vacuum mode and high-vacuum mode.

## Results

### Isolation and characterization of the USP5 mutants

**Figure 1:**
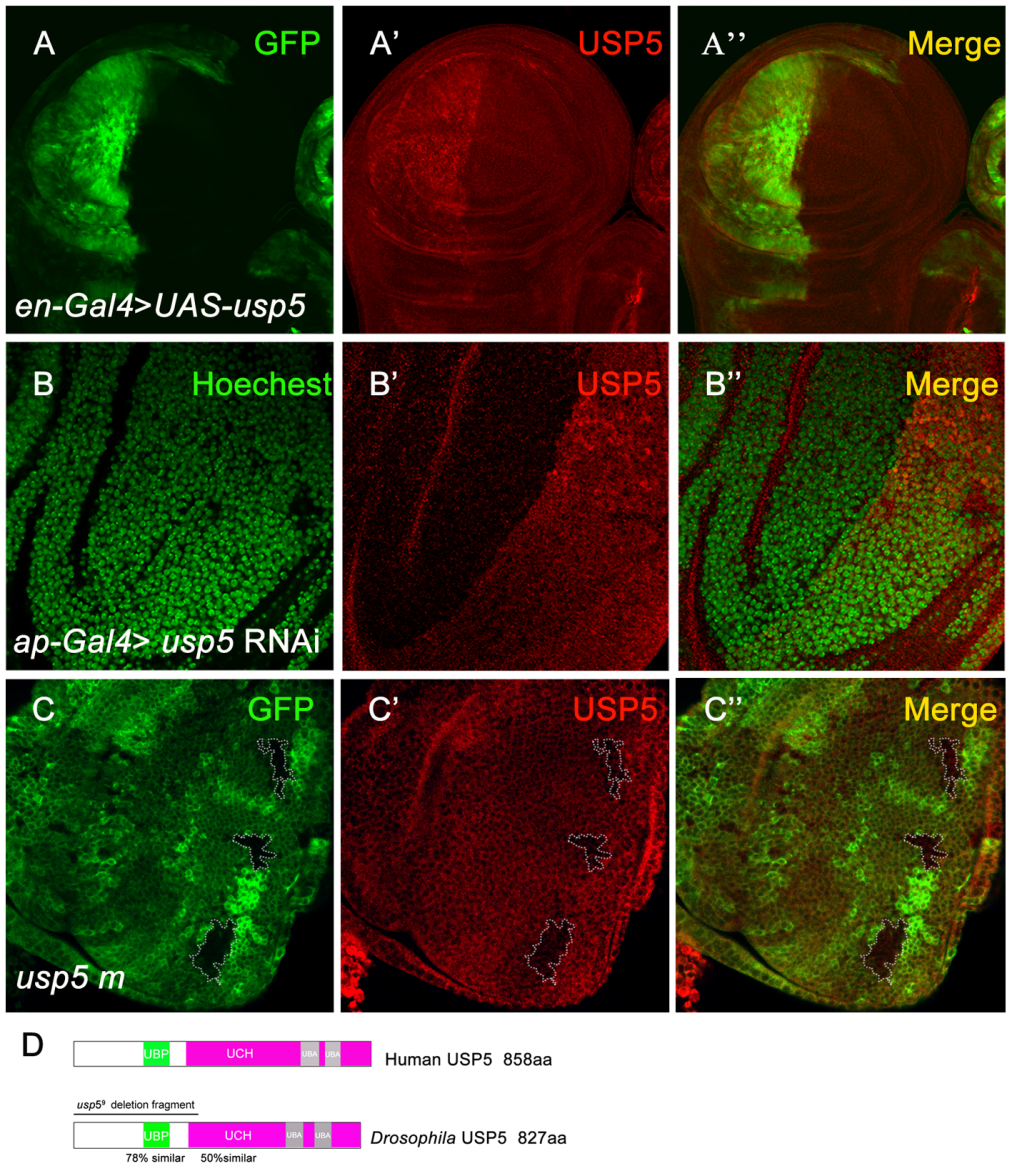
Validation of the *usp5* mutant and antibody. A-A”The specificity of the antibody raised against USP5 was tested by using en-*Gal4* induced ectopic expression *usp5* in the wing disc. The antibody that we made for this study detects over-expression of USP5 A”. B-B” We also tested the antibody specificity for endogenous USP5 protein. *ap-Gal4* induced *usp5* RNAi in the wing disc, and the USP5 protein level was reduced B′. C-C” USP5 was reduced in the *usp5* mutant mosaic clone in eye discs. D Schematic of *Drosophila* USP5 protein shows similarity with human USP5 in the UBP (green) and UCH (purplish red) domain. The straight line shows the *usp5* ^9^ deletion fragment.

To perform a deubiquitinase RNA interference (RNAi) screen, we used *en*-*Gal4* and *ey*-*Gal4* to induce the gene knockdown in the wing and eye in *Drosophila*. We examined phenotypes of adult wings and eyes to determine whether deubiquitinases were involved in wing and eye development in *Drosophila*. We characterized a related, novel *Drosophila* gene, *CG12082*, which encodes a USP family protein and shares 58.58% homology with human USP5 (Figure 1 D). The two independent *usp5* RNAi alleles confirmed that the small eye phenotype is caused by *usp5* knock-down. To further analyze *usp5* gene function, we generated one mutant allele *usp5^9^* by P-element hopping; the *usp5^9^* mutant allele is homozygous lethal. DNA sequencing revealed a deletion mutation in *usp5^9^* that affected the UBP and part of the UCH domain (Figure 1 D). A USP5 antibody confirmed the specificity by overexpression of exogenous USP5*^9^* in wing discs. *En*-*Gal4* was crossed with *UAS*-*usp5* to induce *usp5* overexpression in the posterior compartment of the wing disc. The antibody we developed for this study was sensitive enough to detect the overexpression of USP5 (Figure 1 A′). To test whether this antibody recognized endogenous USP5 protein, we used *ap*-*Gal4* to induce *usp5* RNAi in the dorsal compartment of the wing disc. USP5 staining was reduced following *usp5* knock-down by RNAi (Figure 1 B′). These results show that the USP5 antibody specifically recognizes the USP5 protein. Thus, we next used this antibody to test USP5 protein levels in the *usp5^9^* mutant clone (Figure 1 C′), and found that the protein level was greatly reduced. These data suggest that *usp5^9^* is a null allele for the gene *usp5*. There is another P-element insertion line of *usp5* named *CG12082^EY20760^*, in which the P-element is inserted into the second annotated intron of *usp5*, and the associated lethality is reversed by precise excision of the P-element. *Usp5^9^* and *CG12082^EY20760^* did not complement each other, and exhibited similar phenotypes (data not shown). We therefore used *usp5^9^* to carry out all of the following experiments.

### USP5 is involved in *Drosophila* eye development

**Figure 2:**
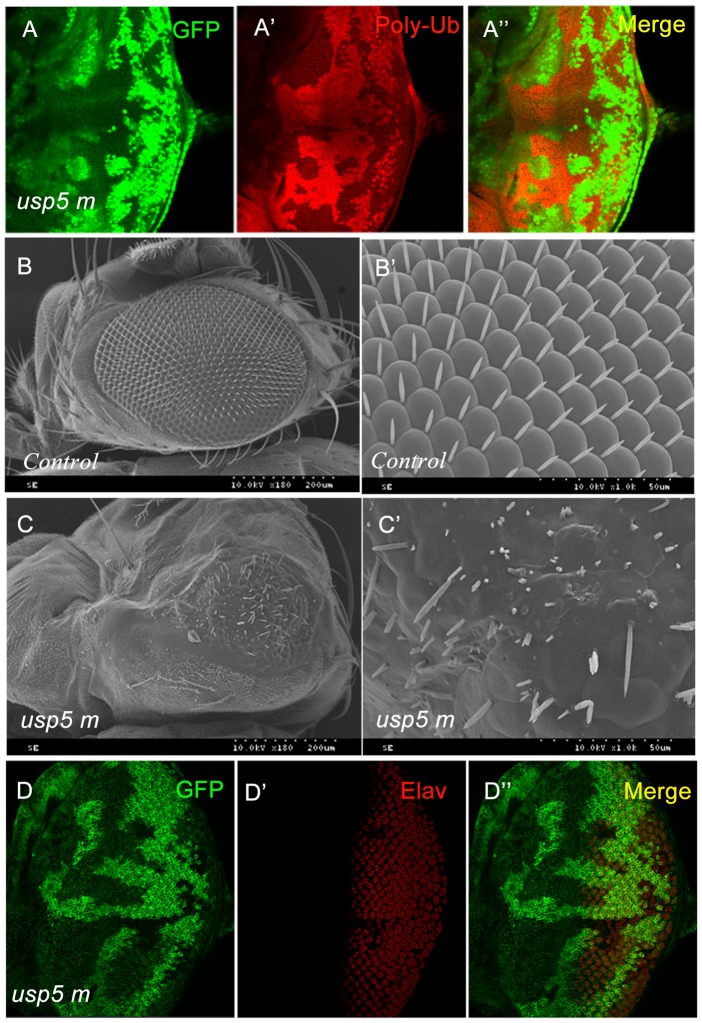
*Usp5* knock-out caused eye defects and poly-ub accumulation. A-A′ The lys48 specific ubiquitin antibody showing that the polyubiquitin chains significantly accumulates in the *usp5* mutant clone. B-B′ Scanning electron micrographs of adult compound eyes. The wild-type (wt) compound eye; magnification×180 (left) and ×1000 (right). C-C′ The *GMR-hid, ey-FLP*, and *usp5^9^* mutants show a small and rough phenotype; magnification×180 (left) and ×1000 (right). D-D′ Elav labeled photoreceptor cells in the eye antennal disc of the third instar larval of the *usp5* mutant clone.

USP5 is known to recycle unanchored polyubiquitin from proteasomes back to free ubiquitin [29]. An *in vitro* study showed that the preferred substrate of USP5 was free/unanchored polyubiquitin chains. The free/unanchored polyubiquitin linked by isopeptide bonds was not conjugated to the substrate protein [24], [30], [31]. The mutation of *ubp14* and the *usp5* homologues in *Saccharomyces cerevisiae, Dictyostelium*, and *Arabidopsis* causes accumulation of free polyubiquitin [32], [33], [34]. In the *usp5* mutant mosaic clones (Figure 2 D), which were marked by no GFP expression, we used the Lys48 specific antibody to label free polyubiquitin chains. The chains accumulated in the *usp5* mutant clone in the eye disc of *Drosophila* (Figure 2 A′), which suggests that the function of *usp5* is conserved.

Accumulation of free polyubiquitin is detrimental since this protein acts as a competitive inhibitor of substrates that bind to the proteasome and other ubiquitin receptors[35]. We are interested in whether the accumulation of free polyubiquitin chains affects *Drosophila* eye development; therefore, we employed the allele for eye-specific expression of *hid* under the GMR promoter to generate an *usp5* homozygous mutant clone in the eye by *ey-FLP/FRT* mediated mitotic recombination. Compared to control fly eyes carrying *FRT-2A* alone (Figure 2 B, B′), the eye of the *usp5* homozygous mutant clone manifested as rough, small, and disordered bristles (Figure 2 C, C′). Furthermore, some of the ommatidias failed to develop in the *usp5* mutant clone eye (Figure 2 C′). Numerous factors are involved in *Drosophila* eye development, including cell signaling, cell death, apoptosis, and protein degradation. To focus on the mechanism behind *usp5* mutant eye defects, we first examined Elav, a photoreceptor cell marker, which is required for correct differentiation and maintenance of the nervous system. We labeled eye discs in the third larval stage with an Elav-specific antibody. In the *usp5* mutant clone, Elav protein level was reduced (Figure 2 D′). This result indicates that the *usp5* gene is involved in *Drosophila* eye development.

### USP5 regulates differentiation of photoreceptors during *Drosophila* eye development

**Figure 3:**
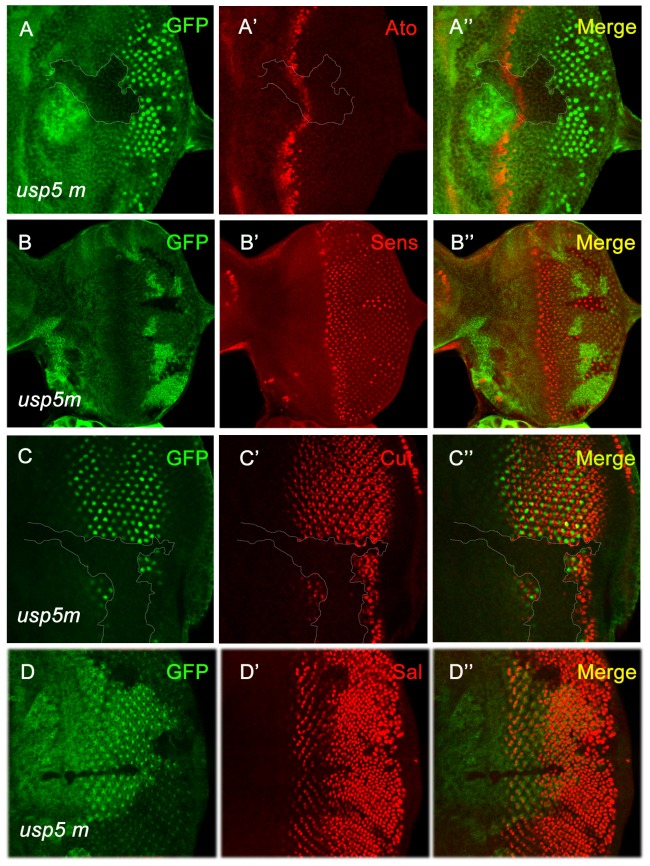
Photoreceptors fail to differentiate in *usp5* mutant eyes. *Usp5* mutant clones were identified by the absence of GFP in all panels. A-A” Ato antibody staining shows delayed Ato expression in the *usp5* mutant clone. B-B” Within the *usp5* mutant clones, the quantity of R8 photoreceptors is reduced. C-C” The cone cells marker Cut is strongly reduced in the *usp5* mutant clones. D-D” The Spalt antibody was used to mark R3/R4 photoreceptors; Spalt expression was reduced in the *usp5* mutant clone.

To identify whether *usp5* mutant clones display eye differentiation defects, we examined important photoreceptor markers during development of the ommatidium. During ommatidium development, the R8 cell forms in the center, around which all photoreceptors are subsequently incorporated into the ommatidia. The key regulator in the specification of R8 is the proneural gene *atonal* (*ato*), a central determinant in peripheral nervous system development [36], [37]. We tested Ato protein levels in the *usp5* mutant using the *minute* clone technique; we found that the Ato was slightly reduced and the morphogenetic furrow (MF) was delayed in the mutant clone compared to wild-type cells. Another factor required for precise R8 specification is Senseless (Sens), which is activated by Ato. Sens is not only required for early R8 selection, but also plays a role in controlling terminal differentiation of the R8 photoreceptor. Sens was used as a photoreceptor R8 marker, so we tested the Sens in *usp5* mutant clones; the amount of R8 photoreceptors was reduced, but R8 photoreceptor survivors showed outgrowth (Figure 3 B′). This illustrates that USP5 is involved in R8 specification and differentiation.

Recruitment of photoreceptors into the advancement ommatidia by the founder R8 follows a stereotypical pattern[38]. The first photoreceptors to be recruited are R2 and R5, followed by R3 and R4, and subsequently R1 and R6. The R3/R4 photoreceptor specification depends on the *spalt* gene complex, which is a target gene of the Dpp pathway. Spalt is also required for R8 photoreceptor terminal differentiation[39]. Our results showed that Spalt was reduced in the *usp5* mutant clone (Figure 3 D′). The eye disc was fully differentiated 42 hours after puparium formation, but it is not known whether *usp5* regulates the differentiation of other cells aside from just photoreceptors. We labeled the eye disc with a Cut antibody and a cone cell marker; this revealed that most cone cells failed to differentiate in the *usp5* mutant clone (Figure 3 C′). Combined, all of the above results indicate that the loss of *usp5* caused defects in the specification and differentiation of eye photoreceptors.

### USP5 regulates the apoptosis pathway in the *Drosophila* eye

**Figure 4:**
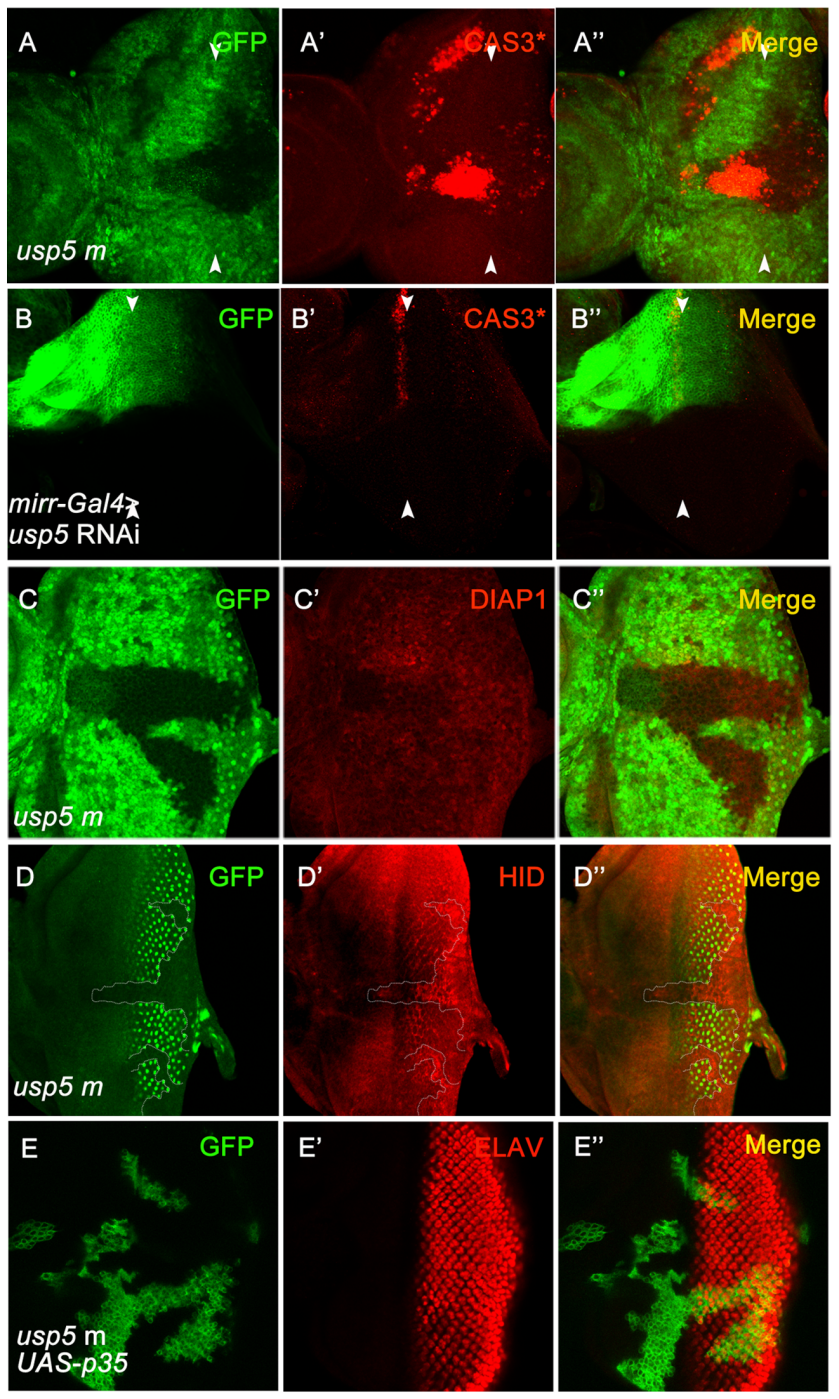
Loss of *usp5* induces apoptosis in the *Drosophila* eye disc. A-A” Caspases accumulated in the *usp5* mutant clone, and the accumulation is greatest anterior to the MF (arrow marked). B, *Mirr-Gal4* induces *usp5* RNAi expression in the dorsal half part of eye discs; GFP labels the *mirr* gene expression position. B′, Caspases are increased when *usp5* is knocked-down. C-C” DIAP1 is reduced in the *usp5* mutant clones in eye discs. D-D” The apoptotic activator HID increased in *usp5* mutant clones. E-E” *p35* is over expressed in the *usp5* mutant clone as determined using MARCM techniques. E Clones marked with GFP. E′ Elav staining was normal in *usp5* mutant clones.

To focus on the mechanisms by which USP5 regulates eye development, we first wanted to know whether apoptosis is induced by the knock-out of *usp5*, since there is a possibility that interference with the differentiation process leads to apoptosis. Apoptosis has occasionally been observed with a rough eye phenotype[40]. We hypothesized that inappropriate activation of DRONC caused the apoptotic phenotype in the *usp5* mutant. To explore this possibility, we tested DRONC in the *usp5* mutant cells of the eye imaginal disc using *minute* technology, and labeled apoptosis using an activated CASPASE-3 (Cas3*) antibody. Consistent with our hypothesis, immunostaining of the eye disc with the anti-active Cas* antibody revealed an increase in apoptotic cells in the *usp5* mutant clone (Figure 4 A′), especially in the clone anterior to MF of the eye imaginal disc. We also targeted *usp5* with RNAi in the eye disc using the *mirr*-*Gal4* line, which is only expressed in the dorsal part of the eye disc and functions relatively late in development. The Cas3* accumulated at extremely high levels in the dorsal region, anterior to MF, in the eye discs of the third larval instar (Figure 4 B′). This suggests that the apoptosis pathway is activated in *usp5* mutant clones.

The caspase DRONC is ubiquitinated by E3 ligase DIAP1. The protein DIAP1 also suppresses the effector caspase drICE and targets drICE for ubiquitination. As Cas3* accumulated in the *usp5* mutant clones, we first speculated that the phenotype was caused by changes in DIAP1 protein levels. We tested DIAP1 levels in the *usp5* mutant clone using a DIAP1 antibody. As expected, DIAP1 protein level was significantly lower in the *usp5* mutant clone, especially in the anterior portion of the eye disc (Figure 4 C′). The inhibition of drICE mediated by DIAP1 could be countered by RPR, HID, and GRIM[41]. We observed that HID protein levels increased in the *usp5* mutant clones (Figure 4 D′). Since HID binds to DIAP1, the accumulation of HID could reduce DIAP1 protein levels. This result shows that apoptosis is activated upstream of HID in the *usp5* mutant eye discs. To identify the photoreceptor defects were caused by inappropriate activation of apoptosis, we employed *p35* to suppress apoptosis; P35 is cleaved and forms a thioester bond with the caspase active site, thereby causing irreversible inhibition of the caspase[42]. Furthermore, p35 effectively inhibits a broad range of active caspases and can block cell death [43]. We used the MARCM system to over-express *p35* in the *usp5* mutant clones. The clones were marked by GFP expression (Figure 4 E). We found that over-expression of *p35* can rescue the Elav-reduced phenotype in the *usp5* mutant clone of the eye disc (Figure 4 E′). Therefore, *usp5* suppression of apoptosis is necessary for the differentiation of photoreceptors.

### The JNK pathway is activated upon loss of *usp5*

**Figure 5:**
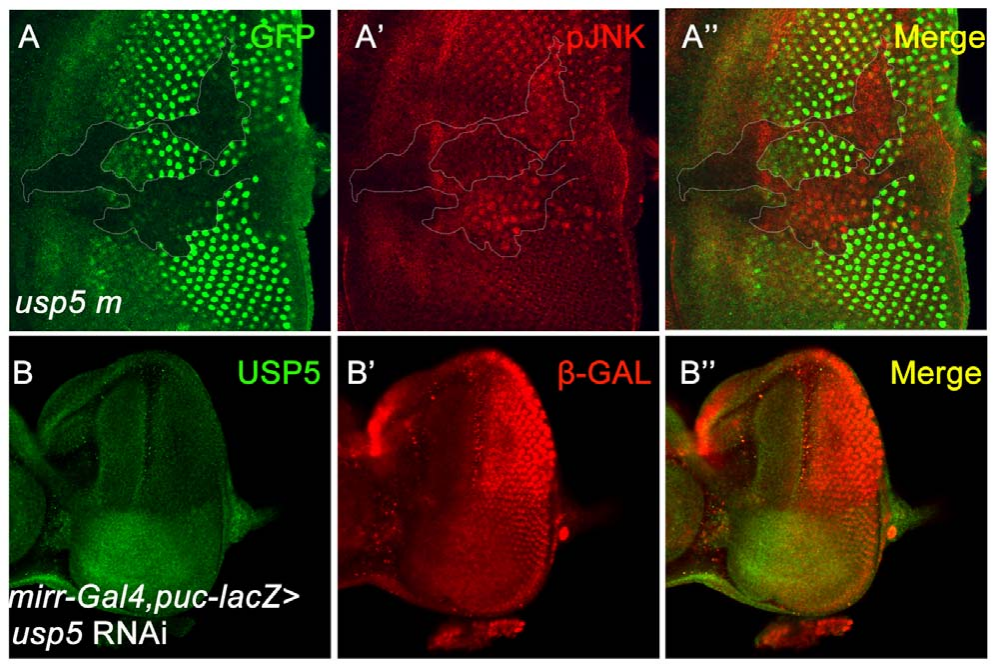
The JNK pathway is activated in the *usp5* mutant clone. A′ Eye imaginal discs from third instar larvae are shown. JNK phosphorylation was used to test the activation of JNK signaling; pJNK is increased in *usp5* mutant clones. B′ *puc* transcription level is labeled using the *puc*-*lacZ* reporter (β-GAL labeling); *puc* level is increased in the dorsal eye imaginal disc where USP5 is knocked-down as driven by *mirr*-*Gal4*.

The JNK pathway appears to be a key component of apoptosis; the JNK and proapoptotic genes establish a positive feedback that amplifies the original apoptotic stimulus [17]. To test whether the JNK pathway is activated by accumulation of DRONC in the *usp5* mutant cells, we first used the pJNK antibody, which recognizes the phosphorylated form of JNK, to test this hypothesis. pJNK level is increased in the *usp5* mutant clone in the eye disc (Figure 5 A′). The activity of JNK was also indicated by the transcriptional activation of *puckered* (*puc*), one of the target genes of JNK signaling in *Drosophila*. *Puc* encodes a JNK-specific phosphatase that down-regulates the pathway, thus forming a negative feedback loop[44]. We used a *mirr-Gal4/puc-lacZ* line to induce *usp5* RNAi expression in the dorsal part of the eye disc. A USP5 antibody was used to detect the effects of RNAi, and anti-β-GAL used to label the *lacZ*. *Puc* transcription levels increased once *usp5* was knocked-down compared to wild-type cells in the same disc (Figure 5 B′). Taken together, these experiments suggest that the JNK pathway is activated once the *usp5* gene is removed.

## Discussion

Our phenotypic characterization of *usp*5 mutants during eye development in *Drosophila* leads to two important conclusions. First, loss of the *usp*5 gene causes severe defects in the specification and differentiation of photoreceptors. Second, loss of *usp*5 activates apoptosis and the JNK pathway.

### Loss of *usp5* causes severe mid-differentiation defects in photoreceptors

Several important factors are involved in regulating eye development in *Drosophila*. Furthermore, specification and differentiation of the photoreceptors are regulated by various cell signaling pathways, cell proliferation, and cell death factors [45]. Some of these pathways, such as the apoptosis pathway[46] and hedgehog pathway[47], are controlled by the ubiquitination-proteasome system. USP5 is able to cleave at least five types of polyubiquitin linkages, including K6, K29, K48, and K63, as well as linear polyubiquitin chains[48]; we therefore anticipated that it regulates eye development. As expected, our data shows that the number of R8 photoreceptors is reduced (Figure 1 C′). The R8 cell serves as the founder cell around which other photoreceptors are subsequently incorporated into the ommatidium. Accompanied by the defect in the photoreceptor R8, the other photoreceptors and cone cells (Figure 3 C′) also showed defects when the *usp5* gene was lost. This is the first time that the in vivo function of USP5 has been demonstrated.

### Loss of *usp5* activates apoptosis and the JNK pathway

The apoptosis pathway plays an important role in *Drosophila* eye development, especially at the end of the process, when most cells survive or die based on the state of their internal caspases. This pathway is controlled by the ubiquitination-proteasome system. The IAP is an E3 ligase that negatively regulates caspases. DIAP1-mediated ubiquitination of DRONC is sufficient for controlling DRONC activation. Furthermore, the DIAP1 antagonist Grim[49] and DIAP1 itself are ubiquitinated by DIAP1. DIAP1 levels are increased in *uba1*, the E1 in *Drosophila* mutant clones [50], [51]. The USP5 serves as a deubiquitinating enzyme, the loss of function of which leads to an accumulation of polyubiquitin chains. Accumulation of free polyubiquitin is probably detrimental to development as it works as a competitive inhibitor of substrate binding to the proteasome[35]. In the *usp5* mutant clone, DIAP1 protein levels were reduced (Figure 4 C′). There are two models to explain this phenotype: 1) the ubiquitination of DIAP1 increased and the stability of DIAP1 declined because of degradation by a proteasome; or, 2)the DIAP1 was inhibited by the upstream antagonists HID, RPR, and GRIM. It has previously been shown that over-expression of *hid*, *rpr*, or *grim* resulted in a loss of DIAP1 in the wing disc and embryo [52]. HID promotes cell death by stimulating DIAP1 auto-ubiquitination and degradation [9], [53]. In the case of the *usp5* mutant, we observed an increase in HID protein level (Figure 4 B′). The reduction of the DIAP1 protein is possibly caused by HID accumulation. These results suggest that *usp5* regulates the apoptosis pathway upstream of HID. More interestingly, the elevation of HID in the posterior compartment did not cause the same accumulation of caspases as in the anterior compartment. A potential explanation for this is that in the mammalian cell caspase-8 is a key initiator of caspase, and the activation is dependent on the polyubiquitination induced by cullin3 (CUL3) -based E3 ligase[54]. The HIB protein functions as substrate recognition subunits for Cul3-based modular ubiquitin ligases, which are expressed only posterior to the MF [55]. The start of caspase activation may also require polyubiquitination induced by CUL3 in *Drosophila*. We found that the expression level of *hib* was strongly reduced in the *usp5* mutant clones of eye disc (unpublished data); this may lead to relatively little caspase accumulation in the posterior of the eye disc.

The p53 and JNK pathways are the major proapoptotic factors in *Drosophila*, playing prominent roles in inducing apoptosis. p53 accumulation is observed when *usp5* is knocked down in the ARN8 cell line[22]. However, we did not observe an accumulation of Dp53 in the *usp5* mutant clones in *Drosophila* eyes or wing discs (data not shown). This suggests that *usp5* may not involve in Dp53 degradation. The JNK signaling also plays an important role in promoting apoptosis in response to a diverse array of signals, including both the p53-dependent response to DNA damage and developmentally regulated apoptosis[56], [57]. JNK-induced up regulation of HID was observed in eye imaginal discs[58]. We observed an increase in phosphorylated JNK in the *usp5* mutant clones. Furthermore, transcription levels of the target gene of the JNK pathway, *puc*, were increased (Figure 5 B′). *Puc* prevents apoptosis by buffering basal levels of JNK signaling. A pilot study showed that the PUC protein is ubiquitinated by the E3 ligase HIB (also called D-SPOP) *in vivo* [59]. Also, PUC protein levels were significantly reduced when co-expressed with D-SPOP in S2 cells [60]. Interestingly, we found that *hib*-*lacZ* was strongly reduced in the *usp5* mutant clones of eye discs (data unpublished). The stability of the HIB protein depends on a high level of hedgehog signaling [55]. The key next step in advancing our knowledge of this system will be to further investigate the precise role of *usp5* in regulating *puc* activity towards JNK and the activation of apoptosis in development. As inappropriate activation of mammalian JNK can lead to various forms of human cancers [61], [62], these types of genetic studies in model organisms contribute to the broader understanding of oncogenic processes in mammals.
